# Magnetic resonance imaging evaluation of cochlear and vestibular nerve calibre: a case-control study in Ménière’s disease and endolymphatic hydrops

**DOI:** 10.1007/s00405-024-08895-4

**Published:** 2024-08-16

**Authors:** Radwa Khalifa, Philip Touska, Irumee Pai, Francesco Padormo, Vicky Goh, Joseph V. Hajnal, Steve E. J. Connor

**Affiliations:** 1 Faculty of Medicine, Helwan University, Cairo, Egypt; 2 School of Biomedical Engineering and Imaging Sciences, King’s College London, London, UK; 3 Department of Radiology, Guy’s and St Thomas’ Hospital, London, UK; 4 Department of Ear, Nose and Throat Surgery, Guy’s and St Thomas’ Hospital, London, UK; 5 Medical Physics, Guy’s and St. Thomas’ NHS Foundation Trust, London, UK; 6 Hyperfine, Inc., Guilford, CT USA; 7 Neuroradiology Department, Ruskin Wing, King’s College Hospital, Denmark Hill, London, SE5 9RS UK

**Keywords:** Magnetic resonance imaging, Endolymphatic hydrops, Ear, Inner, Cochlear nerve, Vestibular nerve, Ménière’s disease

## Abstract

**Purpose:**

To compare the calibre of the cochlear (CN), superior vestibular (SVN) and inferior vestibular (IVN) nerves on magnetic resonance imaging (MRI), both between Ménière’s Disease (MD) ears and clinical controls, and between inner ears with and without endolymphatic hydrops (EH) on MRI.

**Methods:**

A retrospective case–control study evaluated patients undergoing MRI for suspected hydropic ear disease from 9/2017 to 8/2022. The CN, SVN, IVN and facial nerve (FN) diameters and cross-sectional areas (CSA) were measured on T2-weighted sequences whilst EH was evaluated on delayed post-gadolinium MRI. Absolute nerve calibre (and that relative to the FN) in unilateral definite MD ears (2015 Barany criteria) was compared to that in both asymptomatic contralateral ears and clinical control ears. Nerve calibre in ears with severe cochlear and vestibular EH was compared to ears without EH. *t* tests or Wilcoxon signed-rank test/Mann–Whitney *U* test were applied (*p* < 0.001).

**Results:**

173 patients (mean age 51.3 ± 15.1, 65 men) with 84 MD (62 unilateral) and 62 clinical control ears were studied. Absolute and relative CN dimensions were decreased in both MD ears (CSA and diameter) and the contralateral asymptomatic ears (CSA) when compared to clinical controls (*p* < 0.001). Absolute nerve dimensions were reduced in both severe vestibular EH (CN, IVN and SVN) and severe cochlear EH (CN) (*p* < 0.001), however this was not evident when adjusted according to facial nerve calibre.

**Conclusion:**

There is decreased absolute CN calibre in both symptomatic and asymptomatic MD ears as well as ears with severe cochlear and vestibular EH on MRI.

**Supplementary Information:**

The online version contains supplementary material available at 10.1007/s00405-024-08895-4.

## Introduction

Ménière’s disease (MD) is an inner ear disorder characterized by episodic vertigo, low- to mid-frequency hearing loss and fluctuating aural symptoms. MD is usually associated with distension of the endolymphatic compartment of the inner ear at the expense of the surrounding perilymphatic compartment, and this is termed endolymphatic hydrops (EH) [1, 2]. Due to the absence of any definitive audiometric, vestibular, or electro-physiological tests, the diagnosis of MD primarily relies on the subjective reporting of symptoms and audiometry. Since there are variable clinical manifestations and the key symptoms are present in only a minority of patients with early disease, this represents a challenge [3, 4].

The optimization of MRI techniques with delayed post-gadolinium sequences has enabled the in vivo visualization of EH and has offered potential imaging biomarkers for MD [5, 6]. The identification of EH on MRI is now a diagnostic criterion in recent guidelines for MD by the Japan Society for Equilibrium Research [7]. Whilst such gadolinium enhanced MRI is considered safe when performed at low doses (0.1–0.3 mmol/kg) in patients with normal renal function, a causative relationship between gadolinium-based contrast agents and nephrogenic systemic fibrosis is described in patients with renal insufficiency [8], and there is also increasing evidence that multiple gadolinium administrations may result in human brain deposits [9]. Therefore, the development of diagnostic features on MRI sequences without gadolinium administration would be of considerable interest.

Researchers have demonstrated that cochlear and vestibular neural loss is a feature of EH and MD [10–13]. However, only limited studies have investigated the calibre of the cochlear and vestibular nerves in MD on T2w MRI sequences without gadolinium [14, 15]. The potential for changes in cranial nerve calibre to be used as a diagnostic marker should be further explored and may also provide further insight into the pathophysiology of MD.

Therefore, the primary aims of this study were to compare the calibre of the cochlear nerve (CN), superior vestibular nerve (SVN) and inferior vestibular nerve (IVN) in symptomatic MD ears to that in asymptomatic contralateral ears and clinical control ears. Secondary aims were to compare these neural dimensions in ears with and without EH on MRI and to correlate them with the duration of symptoms and audiometric parameters in MD ears.

## Methods

### Patients

**Fig. 1:**
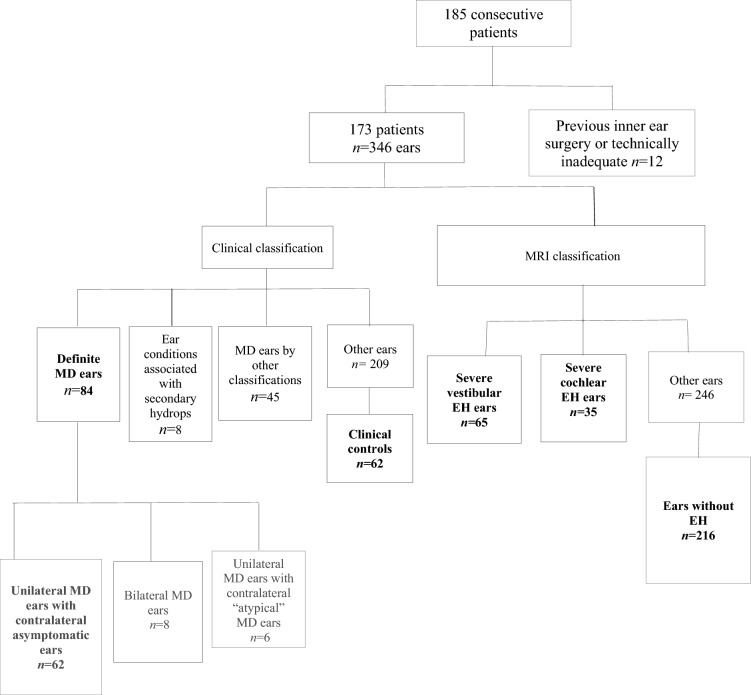
Flowchart demonstrating the selection process for the study cohort

The study was approved by the institutional ethical committee (GSTT Electronic Record Research Interface, IRAS ID: 257,283, Rec Reference: 20/EM/0112). The study included patients with symptoms of hydropic ear disease (episodic vertigo, sudden-onset or fluctuating sensorineural hearing loss (SNHL), aural fullness or tinnitus) who were referred for MRI between September 2017 and August 2022. Exclusion criteria were previous inner ear operations or degraded MR imaging (Table 1; Fig. 1).

**Table 1:** Summary of the demographics of the study cohort

173 patients	
Sex	108 women/65 men
Age	Mean 51.28 ± 15.09 years
MD diagnosis	84 definite MD ears Median 63 (range 1–360)
Air conduction threshold	
Definite MD ears	Mean 56.9 ± 19.6 dBHL
Asymptomatic contralateral MD ears	Mean 23.7 ± 17.7 dBHL

### Imaging technique

All patients were imaged on a 3-T scanner using a 64-channel head and neck coil (Magnetom® Skyra; Siemens Healthcare, Erlangen, Germany). MRI was performed 4 h after the intravenous administration of a double-dose gadolinium-based contrast agent (Gadoterate 0.2 mmol/kg). A T2w SPACE (sampling perfection with application optimized contrasts using different flip angle evolution) (repetition time = 1000 ms, echo time = 125 ms, number of averages = 2, effective refocussing flip angle = 100°, echo train length = 52, pixel spacing = 0.31 mm, slice thickness = 0.3 mm, 262 × 512 matrix, and 160 mm × 80 mm field of view) sequence was used to evaluate neural calibre. An isotropic three-dimensional fluid-attenuated inversion recovery (3D FLAIR) sequence (repetition time = 6000 ms, echo time = 180 ms, inversion time = 2000 ms, number of averages = 1, refocussing flip angle = 180°, echo train length = 27, pixel spacing = 0.7 mm, slice thickness = 0.7, 256 × 240 matrices, 190 mm × 178 mm field of view) was performed to evaluate for EH.

### Clinical data and classification

Two observers (SC, IP) reviewed the contemporary clinical and audiometric data by consensus whilst blinded to imaging findings. Clinical review was always within six months whilst audiometry was performed within 12 months of the MRI study. Definite MD was defined according to the 2015 Barany diagnostic criteria [16] (Supplementary Table 1, Online resource). Definite MD was not diagnosed if there was any ear condition present which was associated with secondary hydrops. Control ears were obtained from patients without definite MD or MD by any previous criteria [8, 17–21] (Supplementary Table 1) and were required to have a) no Meniere’s-type vertigo and b) normal hearing (thresholds ≤ 20 dBHL at 0.5, 1, 2 and 4 kHz) or isolated high frequency sensorineural hearing loss ≥ 20dBHL at ≥ 6 kHz. For the definite MD and asymmetric contralateral MD ears, the mean air conduction threshold (0.25, 0.5, 1, 2, 4, 6, 8 kHz) was recorded from the pure tone audiogram (PTA) performed at the shortest interval from the MRI study. Air conduction (AC) thresholds were used rather than bone conduction (BC) thresholds since the BC thresholds were beyond the limit of the audiometer (worse than moderate/severe hearing loss in a significant proportion of the cases). To ensure that only SNHL was analysed, BC threshold data was collected where obtainable (0.5, 1, 2, 4 kHz). The duration of symptoms (in months) was also recorded for the definite MD cohort.

### Image analysis of neural calibre

**Fig. 2:**
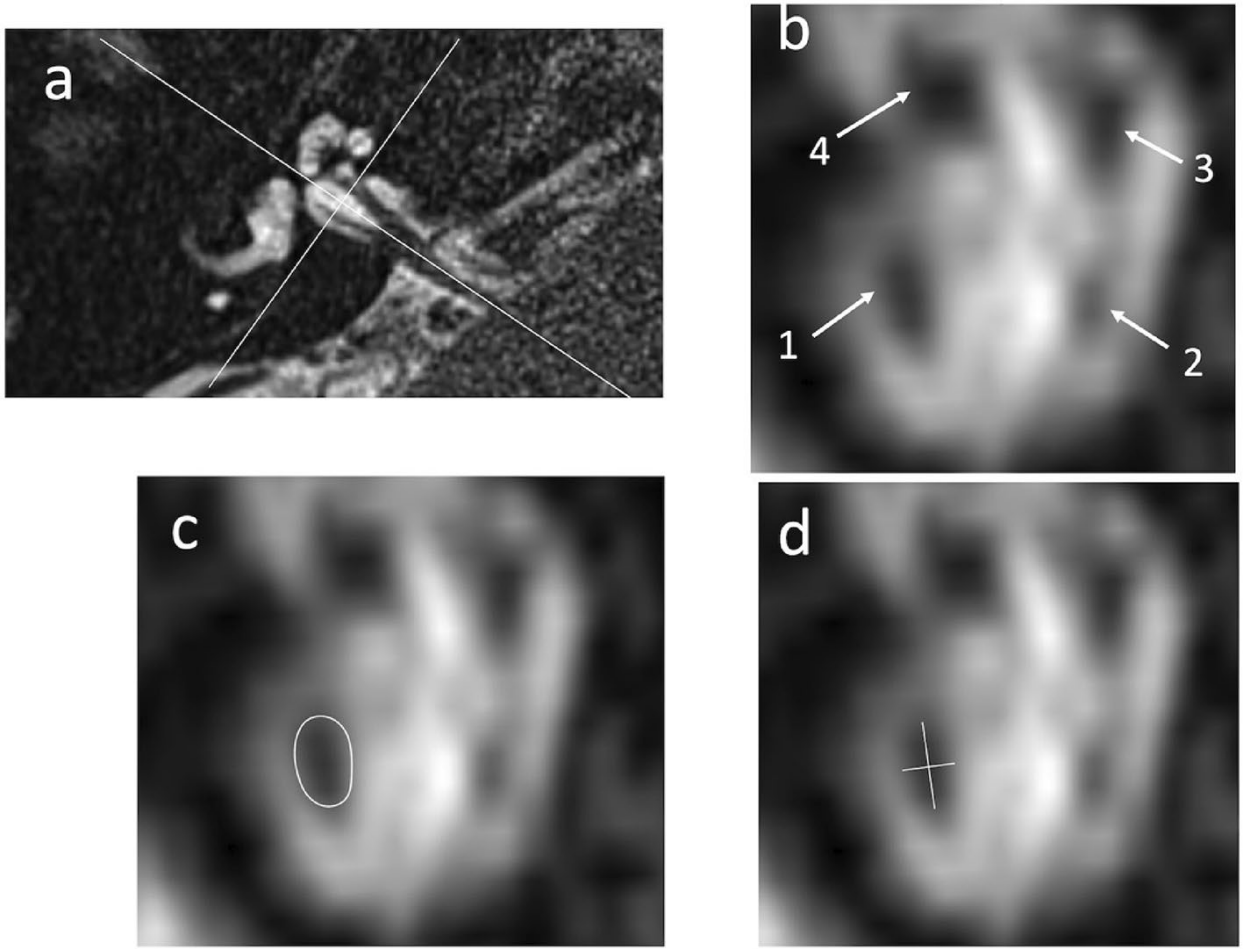
(**a**) Axial T2w SPACE image at the level of the internal auditory canal showing the orientation and location of the oblique sagittal 0.6 mm multiplanar reformat from which neural measurements were obtained (**b**) The corresponding oblique sagittal reformatted image at the fundus of the internal auditory canal is positioned just lateral to the cochlear aperture. The cochlear nerve (1), inferior vestibular nerve (2), superior vestibular nerve (3) and facial nerve (4) are indicated (**c**) The cross-sectional area (CSA) of the antero-inferior cochlear nerve is contoured d) The long diameter (LD) and short diameter (SD) of the cochlear nerve are indicated. Note that the CSA (in **c**) and the LD/SD (in **d**) encompass part of the “penumbra” of the neural outline. The limits were defined as midway between the central neural low signal and the cerebrospinal fluid high signal as imaged with a standardised histogram based PACS algorithm

The cross-sectional measurements of the facial, CN, SVN and IVN were performed for all ears. Analysis was performed on a DICOM-Viewer (Horos v.4.0, Annapolis, MD USA) whilst blinded to clinical diagnosis. Standardised oblique multiplanar reformats (MPRs) were performed perpendicular to a line joining the vestibulocochlear nerve root entry point and the midpoint between the cochlear nerve and inferior vestibular nerve at the fundus of the internal auditory meatus (IAM). To perform neural measurements, an oblique sagittal 0.6 mm slab was reconstructed immediately lateral to the cochlear aperture and displayed with a standardized window width and centre algorithm (Fig. 2). The partial blurring observed at the edges of the reconstructed nerves (penumbra effect) was addressed by contouring at the midpoint of the central low signal and peripheral high signal. The cross-sectional area (CSA), long diameter (LD), and short diameter (SD) of the four nerves were measured on the reformatted 0.6 mm slab (Fig. 2). If the neural course adjacent to the wall of the internal auditory meatus precluded accurate delineation, then this ear was excluded from the analysis. The imaging analysis was performed on two occasions (one month apart) by a single observer (RK with 8 years radiology experience) who was blinded to clinical information. Prior to the study analysis, there was a period of training and joint review of 10 additional cases by SC (28 years of radiology experience).

### Imaging analysis of endolymphatic hydrops and classification

**Fig. 3:**
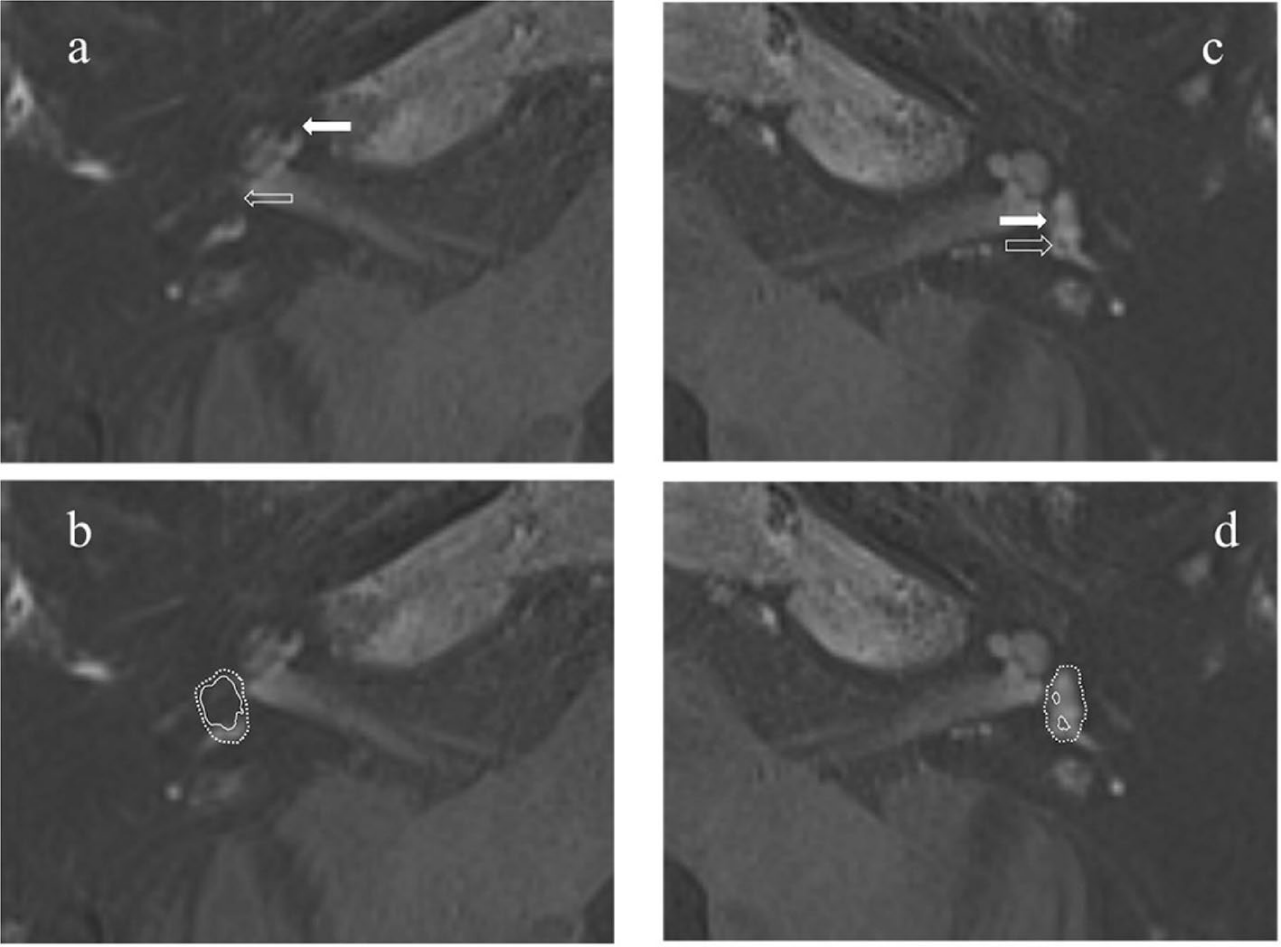
Delayed post-gadolinium 3D FLAIR axial images demonstrate (**a**) Severe vestibulo-cochlear endolymphatic hydrops. Severe cochlear hydrops (solid arrow) and severe vestibular hydrops (open arrow) is indicated by the arrows. **b** The same image demonstrating severe vestibulo-cochlear hydrops with contouring of the vestibular endolymphatic and perilymphatic spaces. The vestibular endolymphatic space comprises the confluent saccule and utricle (central solid contour). This almost replaces the surrounding enhancing vestibular perilymphatic space (peripheral dotted contour) (**c**) Normal endolymphatic appearances. The normal cochlear duct is not perceptible whilst the normal sized non-enhancing saccule (solid arrow) and utricle (open arrow) are clearly visualised within the enhancing perilymphatic space (**d**) The same image demonstrating the normal endolymphatic spaces with contouring of the non-enhancing vestibular endolymphatic space comprising the saccule and utricle (central solid contour). It is surrounded by the enhancing vestibular perilymphatic space which is more clearly visualised (peripheral dotted contour)

For evaluation of the delayed post-gadolinium 3D FLAIR sequence, the imaging plane was reformatted to the orbito-meatal line on a PACS workstation (Sectra workstation, Sectra AB, Sweden), with T2w SPACE images used for anatomical correlation as required. The images were reviewed with standardized magnification and window settings. Two radiologists (PT and SC with 10 and 28 years of radiology experience), independently evaluated the MRI studies. Imaging review was performed whilst blinded to clinical diagnosis and the two observers achieved consensus when different scores were obtained. MRI evidence of “severe” vestibular EH was recorded when there was confluence of the utricle and saccule (Bernaerts grade 2) [22] and “severe” cochlear EH was recorded when there was replacement of the scala vestibuli by the endolymphatic space (Bernaerts and Barath grade 2) with “bands” of low signal endolymph visible within the basal turn [22, 23] (Fig. 3). Control ears were those ears without any features of endolymphatic hydrops on MRI according to any EH grading systems [24].

### Statistical analysis

The data were analysed using SPSS® Statistics 28.0 (IBM^®^, USA. Descriptive statistics were applied to the absolute CN, SVN and IVN dimensions and their ratio to the facial nerve calibre, as well as the audiometry and duration of symptoms. The Kolmogorov–Smirnov test was used to assess whether data was normally distributed with the mean (SD) or median [interquartile range] being documented. The measurements of CN, SVN and IVN calibre for symptomatic MD ears were compared to clinical control ears, whilst those of symptomatic MD ears were compared to contralateral asymptomatic MD ears, and those of contralateral asymptomatic MD ears were compared to clinical control ears. Severe vestibular EH and severe cochlear EH ears were compared to control ears without EH on MRI. Comparisons were performed using the two-sided independent samples *t* test (unpaired data) or paired *t* test (paired data) when normally distributed, whilst the Wilcoxon signed-rank test (paired data) or Mann–Whitney *U* test (unpaired data) were applied when not normally distributed. In view of the multiple comparisons, *p* < 0.001 was considered statistically significant.

Spearman’s rank correlation coefficients evaluated the correlation between nerve calibre measures and AC PTA thresholds for both symptomatic MD ears and contralateral asymptomatic MD ears, whilst paired *t* test compared the AC PTA thresholds between symptomatic MD ears and their contralateral asymptomatic ears. The duration of MD symptoms was also correlated with nerve calibre for symptomatic MD ears.

## Results

### Descriptive statistics

There were 185 consecutive patients referred for delayed post-gadolinium MRI. Patients with degraded MR imaging or inner ear surgery (*n* = 12) were excluded from the study. The final study cohort consisted of 173 patients (108 females, 65 males; age mean 51.28 ± 15.09 years). Of the 346 ears, there were 84 ears classified as definite MD (62 unilateral, 8 bilateral, 6 unilateral with contralateral “atypical” MD ears) [16]) with the median duration of symptoms being 63 months (range 1–360). There were 45 ears fulfilling other clinical criteria for MD [7, 17–21] (Supplementary Table 1) and there were 8 ears with conditions associated with secondary hydrops. Of the remaining ears, there were 62 which satisfied the criteria for clinical controls.

Delayed post-gadolinium MRI demonstrated 65 ears with severe vestibular EH (32 also had severe cochlear EH) whilst 35 ears demonstrated severe cochlear EH (32 also had severe vestibular EH). There were 216 control ears which demonstrated no vestibular or cochlear EH on delayed post gadolinium MRI (Table 1; Fig. 1).

The mean AC threshold of the pure tone audiogram was 56.9 (SD 19.6) for the definite MD ears and 23.7 (SD 17.7) for the asymptomatic contralateral MD ears. There was no mean air bone gap > 15dB at any of the frequencies evaluated. It was not possible to clearly delineate the IVN in 3/346 ears and the FN in 2/346 so these nerves were excluded from the analysis.

### Comparisons of neural calibre in clinical MD ears with controls

The CSA, LD, and SD (absolute and CN/FN ratio) of the CNs were significantly reduced (*p* < 0.001) in ears with symptomatic MD compared to clinical control ears. The SVN and IVN size measurements did not significantly differ between symptomatic MD and clinical control ears (Table 2). No significant differences were observed in the absolute size measurements of CN, SVN, or IVN, or their relative size ratios to FN, when comparing symptomatic MD ears with their asymptomatic contralateral ears (Table 3).

**Table 2:** Comparison of the dimensions of cranial nerves in definite Ménière’s disease ears with control ears

Nerve	*n* =		Parameter	CSA		LD		SD	
	Definite MD	Clinical control		Definite MD	Clinical control	Definite MD	Clinical control	Definite MD	Clinical control
CN	84	62	Mean ± SD *P* value	0.38 ± 0.06 ** < 0.001**	0.51 ± 0.11	0.80 ± 0.15 ** < 0.001**	0.95 ± 0.17	0.58 ± 0.05 ** < 0.001**	0.68 ± 0.09
CN/FN ratio	82	62	Mean ± SD *P* value	1 ± 0.21 ** < 0.001**	10.2 ± 0.29	0.99 ± 0.2 ** < 0.001**	10.1 ± 0.2	0.97 ± 0.11 ** < 0.001**	1 ± 0.16
IVN	83	62	Mean ± SD *P* value	0.33 ± 0.07 0.016	0.36 ± 0.09	0.77 ± 0.12 0.015	0.82 ± 0.12	0.52 ± 0.06 0.126	0.54 ± 0.07
IVN/FN ratio	82	62	Mean ± SD *P* value	0.85 ± 0.22 0.420	0.88 ± 0.18	0.96 ± 0.21 0.027	1 ± 0.23	0.85 ± 0.15 0.582	0.84 ± 0.11
SVN	84	62	Mean ± SD *P* value	0.35 ± 0.08 0.002	0.39 ± 0.08	0.80 ± 0.15 0.008	0.75 ± 0.13	0.55 ± 0.07 0.002	0.59 ± 0.07
SVN/FN ratio	82	62	Mean ± SD *P* value	0.91 ± 0.18 0.126	0.96 ± 0.19	0.98 ± 0.15 0.028	1 ± 0.2	0.92 ± 0.1 0.759	0.93 ± 0.11

*P* value < 0.001 in bold

*CN* cochlear nerve, *CSA* cross-sectional area, *FN* facial nerve, *IVN* inferior vestibular nerve, *LD* long diameter, *MD* Ménière’s disease, *SD* short diameter, *SD* standard deviation, *SVN* superior vestibular nerve

**Table 3:** Comparison of the dimensions of cranial nerves in unilaterally affected Ménière’s Disease ears with their asymptomatic contralateral ears

Nerve	*n* =	Parameter	CSA		LD		SD	
			Unilateral definite MD	Contralateral ear	Unilateral definite MD	Contralateral ear	Unilateral definite MD	Contralateral ear
CN	62	Mean ± SD *P* value	0.38 ± 0.06 0.897	0.38 ± 0.08	0.81 ± 0.08 0.294	0.82 ± 0.11	0.58 ± 0.05 0.160	0.57 ± 0.07
CN/FN ratio	60	Mean ± SD *P* value	1 ± 0.27 0.120	10.05 ± 0.33	1 ± 0.14 0.034	1 ± 0.16	0.99 ± 0.18 0.296	1 ± 0.13
IVN	62	Mean ± SD *P* value	0.33 ± 0.07 0.530	0.32 ± 0.06	0.76 ± 0.12 0.306	0.75 ± 0.08	0.52 ± 0.06 0.530	0.53 ± 0.08
IVN/FN ratio	60	Mean ± SD *P* value	0.85 ± 0.31 0.139	0.89 ± 0.25	0.96 ± 0.02 0.442	0.99 ± 0.01	0.85 ± 0.14 0.020	0.91 ± 0.2
SVN	62	Mean ± SD *P* value	0.35 ± 0.08 0.606	0.34 ± 0.09	0.77 ± 0.1 0.908	0.77 ± 0.11	0.56 ± 0.07 0.410	0.55 ± 0.07
SVN/FN ratio	60	Mean ± SD *P* value	0.92 ± 0.2 0.133	0.93 ± 0.27	0.98 ± 0.14 0.155	1 ± 0.12	0.92 ± 0.09 0.201	0.95 ± 0.1

*CN* cochlear nerve, *CSA* cross-sectional area, *FN* facial nerve, *IVN* inferior vestibular nerve, *LD* long diameter, *MD* Ménière’s disease, *SD* short diameter, *SD* standard deviation, *SVN* superior vestibular nerve

In addition, asymptomatic contralateral MD ears demonstrated a significant reduction (*p* < 0.001) in the absolute CSA, LD and SD of the CN, as well as the CSA CN/FN ratio when compared to clinical control ears. The adjusted SD according to IVN/FN ratio and the absolute SD of the SVN were also significantly decreased (*P* < 0.001) compared to clinical control ears (Table 4).

**Table 4:** Comparison of the dimensions of cranial nerves in the contralateral asymptomatic Ménière’s disease ears with control ears

Nerve	*n* =		Parameter	CSA		LD		SD	
	Non-affected side	Clinical control		Non-affected side	Clinical control	Non-affected side	Clinical control	Non-affected side	Clinical control
CN	62	62	Mean ± SD *P* value	0.38 ± 0.8 ** < 0.001**	0.51 ± 0.11	0.82 ± 0.11 ** < 0.001**	0.95 ± 0.175	0.55 ± 0.095 ** < 0.001**	0.67 ± 0.10
CN/FN ratio	60	62	Mean ± SD *P* value	10.09 ± 0.217 ** < 0.001**	10.26 ± 0.29	10.07 ± 0.24 0.005	10.17 ± 0.23	10.002 ± 0.12 0.012	10.06 ± 0.19
IVN	62	62	Mean ± SD *P* value	0.32 ± 0.06 0.008	0.36 ± 0.09	0.77 ± 0.11 0.004	0.79 ± 0.14	0.52 ± 0.11 0.527	0.52 ± 0.07
IVN/FN ratio	60	62	Mean ± SD *P* value	0.93 ± 0.24 0.334	0.87 ± 0.305	10.015 ± 0.18 0.158	10.05 ± 0.23	0.84 ± 0.11 ** < 0.001**	0.93 ± 0.15
SVN	62	62	Mean ± SD *P* value	0.34 ± 0.09 0.004	0.39 ± 0.08	0.77 ± 0.11 0.04	0.81 ± 0.102	0.54 ± 0.09 ** < 0.001**	0.59 ± 0.122
SVN/FN ratio	60	62	Mean ± SD *P* value	0.94 ± 0.27 0.880	0.95 ± 0.27	10.008 ± 0.18 0.429	10.044 ± 0.204	0.95 ± 0.14 0.268	0.93 ± 0.13

*P* value < 0.001 in bold

*CN* cochlear nerve, *CSA* cross-sectional area, *FN* facial nerve, *IVN* inferior vestibular nerve, *LD* long diameter, *SD* short diameter, *SD* standard deviation, *SVN* superior vestibular nerve

### Comparisons of neural calibre in endolymphatic hydrops ears with controls

The absolute CSA, LD, and SD of the CN were significantly decreased in ears with severe vestibular EH compared to control ears without EH on MRI (*p* < 0.001). However, this was not maintained when the CN dimensions were adjusted according to the CN/FN ratio. Similarly, the absolute CSA and SD of both the IVN and SVN were significantly decreased in severe vestibular EH (*p* < 0.001) compared to control ears, whilst the IVN/FN and SVN/FN calibre ratios did not significantly differ. The absolute CSA and SD of the CN were significantly decreased (*p* < 0.001) in ears with severe cochlear EH compared to control ears without EH on MRI, but again there was no significant reduction in the CN/FN ratio. The SVN and IVN size measurements did not significantly differ between severe cochlear EH control ears and control ears without EH on MRI (Tables 5 and 6).

**Table 5:** Comparison of the dimensions of cranial nerves in ears with severe vestibular endolymphatic hydrops with control ears

Nerve	*n* =		Parameter	CSA		LD		SD	
	Vestibular EH	MRI control		Vestibular EH	MRI control	Vestibular EH	MRI control	Vestibular EH	MRI control
CN	65	216	Mean ± SD *P* value	0.39 ± 0.1 ** < 0.001**	0.47 ± 0.18	0.81 ± 0.09 ** < 0.001**	0.89 ± 0.14	0.58 ± 0.07 ** < 0.001**	0.64 ± 0.152
CN/FN ratio	65	216	Mean ± SD *P* value	10.01 ± 0.28 0.013	10.1 ± 0.34	10.04 ± 0.22 0.089	10.09 ± 0.23	0.99 ± 0.11 0.034	10.02 ± 0.16
IVN	65	216	Mean ± SD *P* value	0.32 ± 0.09 ** < 0.001**	0.37 ± 0.13	0.77 ± 0.15 0.02	0.82 ± 0.16	0.51 ± 0.09 ** < 0.001**	0.57 ± 0.11
IVN/FN ratio	65	216	Mean ± SD *P* value	0.87 ± 0.2 0.181	0.91 ± 0.23	10.01 ± 0.25 0.82	10.007 ± 0.2	0.85 ± 0.14 0.026	0.9 ± 0.16
SVN	65	216	Mean ± SD *P* value	0.34 ± 0.1 ** < 0.001**	0.39 ± 0.15	0.76 ± 0.12 0.002	0.81 ± 0.16	0.55 ± 0.06 ** < 0.001**	0.6 ± 0.09
SVN/FN ratio	65	216	Mean ± SD *P* value	0.91 ± 0.19 0.262	0.95 ± 0.28	0.99 ± 0.17 0.291	10.01 ± 0.2	0.95 ± 0.12 0.28	0.94 ± 0.16

*P* value < 0.001 in bold

*CN* cochlear nerve, *CSA* cross-sectional area, *EH* endolymphatic hydrops, *FN* Facial nerve, *IVN* Inferior vestibular nerve, *LD* long diameter, MRI magnetic resonance imaging, *SD* short diameter, *SD* standard deviation, *SVN* superior vestibular nerve

**Table 6:** Comparison of the dimensions of cranial nerves in ears with severe cochlear endolymphatic hydrops with control ears

Nerve	*n* =		Parameter	CSA		LD		SD	
	Cochlear EH	MRI control		Cochlear EH	MRI control	Cochlear EH	MRI control	Cochlear EH	MRI control
CN	35	216	Mean ± SD *P* value	0.39 ± 0.06 ** < 0.001**	0.46 ± 0.18	0.82 ± 0.13 0.022	0.88 ± 0.19	0.57 ± 0.07 ** < 0.001**	0.64 ± 0.15
CN/FN ratio	35	216	Mean ± SD *P* value	10.01 ± 0.26 0.121	10.104 ± 0.34	10.06 ± 0.21 0.525	10.09 ± 0.23	0.97 ± 0.18 0.086	10.02 ± 0.16
IVN	35	216	Mean ± SD *P* value	0.32 ± 1 0.016	0.37 ± 0.13	0.78 ± 0.14 0.350	0.8 ± 0.16	0.52 ± 0.08 0.002	0.57 ± 0.11
IVN/FN ratio	35	216	Mean ± SD *P* value	0.87 ± 0.27 0.914	0.89 ± 0.29	10.02 ± 0.27 0.294	10.007 ± 0.206	0.85 ± 0.14 0.113	0.905 ± 0.16
SVN	35	216	Mean ± SD *P* value	0.32 ± 0.1 0.006	0.37 ± 0.13	0.78 ± 0.14 0.045	0.8 ± 0.16	0.52 ± 0.08 0.006	0.57 ± 0.11
SVN/FN ratio	35	216	Mean ± SD *P* value	0.98 ± 0.28 0.942	0.95 ± 0.28	10.01 ± 0.19 0.802	10.017 ± 0.2	0.93 ± 0.013 0.315	0.95 ± 0.12

*P* value < 0.001 in bold

*CN* Cochlear nerve, *CSA* cross-sectional area, *EH* endolymphatic hydrops, *FN* Facial nerve, *IVN* Inferior vestibular nerve, *LD* long diameter, *MRI* magnetic resonance imaging, *SD* short diameter, *SD* standard deviation, *SVN* Superior vestibular nerve

### Comparisons of pure tone audiometry and disease duration with neural calibre in MD patients

There was a positive correlation (*r* = 0.27, *p* < 0.05) between the mean AC threshold and the absolute CSA of the CN for the asymptomatic contralateral MD ears but not for the symptomatic MD ears (Supplementary Table 2, Online Resource). The symptomatic MD ears showed a significantly higher mean AC threshold compared to their asymptomatic contralateral ears (*P* < 0.05). The neural dimensions did not significantly correlate with symptom duration in the definite MD ears (Supplementary Table 3, Online Resource).

### Reliability statistics

The intra-rater reliability for nerve size measurements was calculated for all ears where the nerves could be delineated (Supplementary Table 4, Online Resource). The reliability of CSA measurements (0.664–0.884) was superior to that of SD (0.535–0.633) and LD (0.516–0.653). The IVN CSA demonstrated the highest kappa value of κ = 0.814 (95% CI 0.744–0.883) with very good agreement. The poorest agreement was recorded for the LD of the FN with a κ = 0.516 (95% CI 0.295–0.738). The intra-rater reliability for CN CSA was κ = 0.715(CI 0.589–0.842) with substantial agreement. The inter-rater reliability for the evaluation of severe cochlear EH was κ = 0.858 whilst that for vestibular EH was κ = 0.941 with very good agreement.

## Discussion

The MRI based measurements of cochlear nerve were significantly reduced in symptomatic definite Ménière’s disease (MD) ears compared to the clinical control ears, even when scaled to the facial nerve calibre as a reference (*p* < 0.001). However vestibular nerve calibre did not differ between symptomatic MD ears and clinical control ears. Cochlear and vestibular nerve dimensions did not significantly differ between the symptomatic MD ears and their contralateral asymptomatic ears. Whilst all cochlear nerve dimensions were significantly reduced in the asymptomatic ears of MD patients as compared to clinical control ears (*p* < 0.001), this was only maintained for the cross-sectional area (CSA) when adjusted to the facial nerve calibre. In ears with severe vestibular endolymphatic hydrops on delayed post-gadolinium MRI, the cochlear and vestibular nerve CSAs were significantly reduced when compared to ears without endolymphatic hydrops (*p* < 0.001); whilst in the presence of severe cochlear endolymphatic hydrops only the cochlear nerve CSA was significantly decreased. There was no reduction in neural calibre with either severe vestibular or cochlear EH when it was referenced to the facial nerve. The cochlear nerve CSA did not demonstrate a significant correlation with the mean air conduction thresholds on pure tone audiometry or with the duration of symptoms in ears with definite MD.

The only previous study to analyse CN, SVN, IVN dimensions in patients with MD was conducted by Henneberger et al. [14]. Their observation of increased CSA measurements in the CN, SVN, IVN and FN in both symptomatic and asymptomatic ears of MD patients contrast with our findings. There are methodological differences which may explain this disparity. Firstly, our study included a larger sample size with 84 MD and 62 clinical control ears as compared to 21 and 43 ears respectively, so the study was less prone to type I and type II errors. Secondly, we accounted for constitutional differences in neural dimensions by using the facial nerve dimensions as a reference [25, 26]. Interestingly, our findings of absolute reductions in cranial nerve size in the setting of severe EH on MRI was not confirmed when the ratio to facial nerve size was calculated. Thirdly, our study incorporated clinical criteria for MD according to the latest 2015 Barany Society criteria [16]. Fourthly, although Henneberger et al., [14] confirmed the presence of endolymphatic hydrops in their cohort of MD ears, our additional evaluation of the presence of endolymphatic hydrops in control ears allowed a secondary analysis according to the presence of endolymphatic hydrops. This is of importance since severe endolymphatic hydrops has been demonstrated on MRI in up to 27% of clinical control ears [27]. Finally, our key findings with regards to the CN CSA were supported by intra-rater reliability data confirming substantial agreement.

The finding of reduced absolute cochlear nerve calibre in ears with endolymphatic hydrops is in accordance with a previous animal study. Megerian et al. [10] demonstrated that inducing EH in a guinea pig model resulted in a significant reduction in CN maximal diameters. It was proposed that neuronal injury was the primary mediator of EH-related sensorineural hearing loss, and this has been supported by other studies showing a reduced number of afferent nerve endings and their synaptic contacts in the presence of EH [28]. This suggests a potential for MRI evaluation of cochlear nerve dimensions to act as a clinically significant biomarker for neural damage resulting from EH. In addition, the finding of decreased contralateral CN dimensions in the asymptomatic ears of patients with MD suggests that neural loss is occurring prior to clinical presentation, and it raises the possibility of whether this finding could have prognostic implications. Evidence for its pathogenesis is provided by Kariya et al. [29] who demonstrated loss of spiral ganglion cells in the temporal bone of the asymptomatic ear in MD patients.

We had hypothesized that the variation in neural loss between the CN, SVN, IVN and FN may be a useful diagnostic marker of MD that could be derived from MRI without gadolinium enhancement. Unfortunately, the finding of reduced CN CSA alone is a non-specific finding since MRI has demonstrated CN “deficiency” due to hypoplasia or acquired volume loss in a range of sensorineural hearing disorders [30, 31]. Nevertheless, CN deficiency would be important to evaluate prior to considering cochlear implantation for MD patient, since it impacts on post implant auditory performance, and hence its identification aids both surgical decision making and pre-operative counselling [32, 33]. The apparent preservation of vestibular nerve calibre is of interest since loss of Scarpa’s ganglion cells [12] and vestibular nerve fibres [11] have both been recorded in temporal bones with documented MD.

Nakamichi et al. [34] and Jaryszak et al. [35] used a similar methodology for measuring the CN near the fundus of the internal auditory meatus in normal hearing ears. It is of interest that the CN CSAs recorded in these studies (1.07 ± 0.30 mm^2^ [34] and 1.1 ± 0.26 mm^2^ [35]) were increased relative to those of the normal hearing controls in this study (0.51 ± 0.11). One explanation for this discrepancy is that these previous studies derived CSA from a formula based on the linear dimensions whereas our methodology involved tracing the contour of the nerve periphery. The difference in findings may also be attributed to variations in MR sequences, window setting algorithm and the degree of “penumbra” included. This finding does not undermine the validity of our comparison between study groups since MRI parameters and methodology were consistent.

Limitations of this study should be addressed. Firstly, the retrospective nature of the study led to a variation in the time interval between the MRI and the clinical MD reference standard. Secondly, with respect to patient selection, there is inherent bias due to the case–control design [36] whilst there is potential criticism of the control group since some experienced audio-vestibular symptoms. Thirdly, most of the severe endolymphatic hydrops cohort demonstrated vestibulo-cochlear hydrops, so it was difficult to separate the individual effects of vestibular and cochlear endolymphatic hydrops on the neural dimensions. This was particularly the case for severe cochlear hydrops with severe vestibular hydrops being present in 33/36 cases. Fourthly, methodological weaknesses include the absence of interobserver agreement reliability evaluation. Although intra-observer reliability was recorded, some of the linear dimensions demonstrated only moderate agreement which was likely due to variable interpretation of the “penumbra” when contouring the nerves. It is appreciated that there are potential effects of constitutional differences (e.g. age, gender, weight) on nerve calibre which may influence outcomes, but it was attempted to address this by referencing to the ipsilateral facial nerve calibre. Finally, the generalizability of the measurements may be restricted due to their dependence on the specific MRI sequence and technique used in this study.

In conclusion, the cochlear nerve cross sectional area was reduced in both the symptomatic and asymptomatic ears of definite MD patients. A reduction in cochlear nerve calibre was also observed in ears with severe cochlear and vestibular endolymphatic hydrops on MRI, however this reduction was not maintained after scaling to the facial nerve calibre, so these findings should be interpreted with some caution. Future investigations should focus on imaging of neural loss in MD ears and its potential prognostic implications.

## Supplementary Information

Below is the link to the electronic supplementary material.

Supplementary file1 (DOCX 25 KB)

## Abbreviations

3D FLAIR: Three-dimensional fluid-attenuated inversion recovery
AC: Air conduction
BC: Bone conduction
CN: Cochlear nerve
CSA: Cross-sectional area
EH: Endolymphatic hydrops
FN: Facial nerve
IAM: Internal auditory meatus
IVN: Inferior vestibular nerve
LD: Long diameter
MD: Ménière’s disease
MPRs: Multiplanar reformats
MRI: Magnetic resonance imaging
PTA: Pure tone audiogram
SD: Short diameter
SD: Standard deviation
SNHL: Sensorineural hearing loss
SPACE: Sampling perfection with application optimized contrasts using different flip angle evolution
SVN: Superior vestibular nerve

## Declarations

### Guarantor

The scientific guarantor of this publication is Dr Steve Connor.

### Methodology

Retrospective, case–control study, performed at one institution.

### Statistics and biometry

One of the authors has significant statistical expertise.
