# Supplementary material for: Magnetic resonance imaging evaluation of cochlear and vestibular nerve calibre: a case-control study in Ménière’s disease and endolymphatic hydrops
Source: Eur Arch Otorhinolaryngol. 2024 Aug 16;282(1):91–101. doi: 10.1007/s00405-024-08895-4 (PMC11735575; doi:10.1007/s00405-024-08895-4)
Supplement: Supplementary file 1 — Supplementary file1 (DOCX 25 KB) [file 405_2024_8895_MOESM1_ESM.docx]

**Title:**

**Magnetic resonance imaging evaluation of cochlear and vestibular nerve calibre: A case-control study in Ménière’s disease and endolymphatic hydrops**

**European Archives of Otolaryngology**

**Author Information:**

**Radwa Khalifa, Philip Touska, Irumee Pai, Francesco Padormo, Vicky Goh, Joseph V Hajnal, Steve EJ Connor**

Corresponding author: Dr S.E.J. Connor: Kings College Hospital, London, United Kingdom

e mail [steve.connor@kcl.ac.uk](mailto:steve.connor@kcl.ac.uk)

| Clinical classification | Clinical criteria |
| --- | --- |
| *Definite 2015 Barany Society clinical Criteria for MD* [16] | |
|  | A. ≥2 spontaneous episodes of vertigo, each lasting 20 minutes to 12 hours  B. Audiometrically documented low- to medium-frequency SNHL in affected ear on ≥1 occasion before, during or after 1 of the episodes of vertigo  C. Fluctuating aural symptoms (hearing, tinnitus or fullness) in affected ear  D. Not better accounted for by another vestibular diagnosis |
|  |  |
| *Other clinical criteria for MD or hydropic ear disease (“Atypical” MD)* | |
| 2015 MD Criteria [16] |  |
| Probable | A. ≥2 episodes of vertigo or dizziness, each lasting 20 minutes to 24 hours  B. Fluctuating aural symptoms (hearing, tinnitus or fullness) in affected ear  C. Not better accounted for by another vestibular diagnosis |
| 1995 MD Criteria (19) |  |
| Certain Meniere's disease | Definite Meniere's disease, plus histopathologic confirmation |
| Definite Meniere's disease | A. Two or more definitive spontaneous episodes of vertigo 20 minutes or longer  B. Audiometrically documented hearing loss on at least one occasion  C. Tinnitus or aural fullness in the treated ear  D. Other causes excluded |
| Probable Meniere's disease | A. One definitive episode of vertigo  B. Audiometrically documented hearing loss on at least one occasion  C. Tinnitus or aural fullness in the treated ear  D. Other causes excluded |
| Possible Meniere's disease | A. Episodic vertigo of the Meniere type without documented hearing loss, or  B. Sensorineural hearing loss, fluctuating or fixed, with dysequilibrium but without definitive episodes  C. Other causes excluded |
| Monosymptomatic cochlear hydrops (cMD) |  |
| 1972 Criteria (17) | Characterized solely by a fluctuating and progressive  sensorineural deafness with all auditory test results typical of  MD. Many patients notice a fullness in the ear  coincident with the sudden drop in hearing. Some subsequently  develop definitive dizzy spells, and the qualifying ‘‘cochlear’’ is discarded |
| Kimura et al (21) | Fluctuating hearing loss with single episode of vertigo, unsteadiness, or no vestibular symptoms |
| 2021 Japanese Clinical Practice Guideline of Meniere’s Disease (7) | Recurrent cochlear symptoms (eg hearing loss, tinnitus, aural fullness) without vertigo attacks with audiometrically demonstrated SNHL (usually low frequency or pan-frequency) |
| Gurkov (20) | Acute onset low tone acute low tone sudden onset SNHL |
| Summary criteria for cochlear hydrops applied | Fluctuating hearing loss or acute low tone sudden onset SNHL with or without aural fullness/tinnitus but no MD type vertigo (eg only single attack of vertigo alone, or with concurrent unsteadiness). |
| Monosymptomatic vestibular hydrops (vMD) |  |
| 1972 Criteria (17) | Characterized solely by definitive spells of vertigo. This is more  difficult to diagnose as there are no objective findings between spells. The diagnosis may be accepted upon exclusion of other  diseases. Some patients subsequently develop deafness, and the  qualifying ‘‘vestibular’’ is dropped |
| Kimura et al (21) | Recurrent episodic vertigo with or without fixed hearing loss |
| 2021 Japanese Clinical Practice Guideline of Meniere’s Disease (8) | Recurrent attacks of vertigo typical for MD and signs of peripheral dysfunction without accompanying fluctuating cochlear symptoms |
| Summary criteria for vestibular hydrops applied | MD type vertigo/episodic vertigo with or without aural fullness/tinnitus and with or without fixed SNHL (but not fluctuating or low frequency). |
| Control ears | |
|  |  |
|  | Neither ear satisfying clinical criteria for MD on any previous classification or any features of hydropic ear disease (as above) |
|  | Audiogram: Normal thresholds (between 0-25dB at all frequencies) or isolated high frequency hearing loss (>6K) |
|  | No MD type vertigo (recurrent episodic and lasting 20 minutes to 24 hours) |
|  | Aural fullness or tinnitus permitted if it could be explained by an alternative diagnosis (e.g eustachian tube dysfunction) |

MD = Ménière’s disease; SNHL = sensorineural hearing loss; vMD=vestibular Ménière’s disease; cMD= Ménière’s disease

**Supplementary Table 1:** Clinical criteria used to define MD and control ears

| Study group | Nerve | Parameter | CSA (mm^2^ ) | LD (mm) | SD (mm) |
| --- | --- | --- | --- | --- | --- |
| Definite MD ears | CN | r  *P* value | .085  .443 | .162  .140 | -.023  .833 |
| Definite MD ears | CN/FN | r  *P* value | .215  .053 | .141  .205 | .132  .236 |
| Contralateral asymptomatic ears | CN | r  *P* value | **.276**  **.030** | .175  .172 | .0215  .094 |
| Contralateral asymptomatic ears | CN/FN | r  *P* value | .161  .211 | .158  .221 | .058  .652 |

CN=Cochlear nerve; CSA = cross-sectional area; FN= Facial nerve; LD = long diameter; SD = short diameter

**Supplementary Table 2:** The Spearman correlation coefficients (r) and corresponding *P* values between dimensions of cochlear nerve, and mean air conduction on pure tone audiometry within definite Ménière’s disease ears and contralateral asymptomatic ears. *P* value≤0.05 in bold

| Nerve | Parameter | CSA (mm2 ) | LD (mm) | SD (mm) |
| --- | --- | --- | --- | --- |
| CN | r  *P* value | .081  .481 | .118  .296 | .03  .794 |
| IVN | r  *P* value | . 024  .834 | .043  .707 | -.016  .887 |
| SVN | r  *P* value | .215  .055 | .194  .084 | .187  .096 |

CN= Cochlear nerve; CSA= cross-sectional area; IVN= Inferior vestibular nerve; LD= long diameter, SD= short diameter; SVN= Superior vestibular nerve

**Supplementary Table 3:** The Spearman correlation coefficients (r) and corresponding *P* values between dimensions of cranial nerves, and duration of symptoms within definite Ménière’s disease ears

| Nerve | *n=* | CSA (mm2) | LD (mm) | SD (mm) |
| --- | --- | --- | --- | --- |
| CN | 346 | *κ* =0.715(0.589-0.842) | ***κ* =0.557 (0.228-0.687)** | *κ*=0.616(0.420-0.812) |
| IVN | 343 | *κ* =0.814 (0.744-0.883) | *κ* =0.617 (0.394-0.841) | *κ*=0.633(0.467-0.799) |
| SVN | 346 | *κ* =0.664 (0.550-0.779) | *κ* =0.653(0.506-0.799) | ***κ* =0.535 (0.216-0.654)** |
| FN | 344 | *κ* =0.739 (0.599-0.879) | ***κ* =0.516(0.295-0.738)** | *κ*=0.607(0.408-0.806) |

CN= Cochlear nerve; CSA= cross-sectional area; FN= Facial nerve; IVN= Inferior vestibular nerve; LD= long diameter; SD=short diameter; SVN= Superior vestibular nerve

**Supplementary Table 4:** Intra-observer reliability Kappa values for the cranial nerves’ measurements. Kappa values <0.6 in bold
